# "EMMA Study: a Brazilian community-based cohort study of stroke mortality and morbidity": "Estudo EMMA: estudo coorte brasileiro baseado na comunidade sobre mortalidade e morbidade por acidente vascular cerebral"

**DOI:** 10.1590/1516-3180.2016.024227092016

**Published:** 2016-06-03

**Authors:** Alessandra Carvalho Goulart

**Affiliations:** I MD, PhD. Clinical Epidemiologist and Researcher, Center for Clinical and Epidemiological Research, Hospital Universitário, Universidade de São Paulo (HU-USP), São Paulo (SP), Brazil.

**Keywords:** Stroke, Public health surveillance, Cohort studies, Risk factors, Mortality, Acidente vascular cerebral, Vigilância em saúde pública, Estudos coortes, Fatores de risco, Mortalidade

## Abstract

**CONTEXT AND OBJECTIVE:**

Stroke has a high burden of disability and mortality. The aim here was to evaluate epidemiology, risk factors and prognosis for stroke in the EMMA Study (Study of Stroke Mortality and Morbidity).

**DESIGN AND SETTINGS:**

Prospective community-based cohort carried out in Hospital Universitário, University of São Paulo, 2006-2014.

**METHODS:**

Stroke data based on fatal and non-fatal events were assessed, including sociodemographic data, mortality and predictors, which were evaluated by means of logistic regression and survival analyses.

**RESULTS:**

Stroke subtype was better defined in the hospital setting than in the local community. In the hospital phase, around 70% were first events and the ischemic subtype. Among cerebrovascular risk factors, the frequency of alcohol intake was higher in hemorrhagic stroke (HS) than in ischemic stroke (IS) cases (35.4% versus 12.3%, P < 0.001). Low education was associated with higher risk of death, particularly after six months among IS cases (odds ratio, OR, 4.31; 95% confidence interval, CI, 1.34-13.91). The risk of death due to hemorrhagic stroke was greater than for ischemic stroke and reached its maximum 10 days after the event (OR: 3.31; 95% CI: 1.55-7.05). Four-year survival analysis on 665 cases of first stroke (82.6% ischemic and 17.4% hemorrhagic) showed an overall survival rate of 48%. At four years, the highest risks of death were in relation to ischemic stroke and illiteracy (hazard ratio, HR: 1.83; 95% CI: 1.26-2.68) and diabetes (HR: 1.45; 95% CI: 1.07-1.97). Major depression presented worse one-year survival (HR: 4.60; 95% CI: 1.36-15.55).

**CONCLUSION:**

Over the long term, the EMMA database will provide additional information for planning resources destined for the public healthcare system.

## RESUMO

**CONTEXTO E OBJETIVO:**

O acidente vascular cerebral (AVC) tem alta carga de incapacidade e mortalidade. Objetivou-se avaliar a epidemiologia, fatores de risco e prognóstico do AVC no Estudo EMMA (Estudo da Mortalidade e Morbidade do AVC).

**TIPO DE ESTUDO E LOCAL:**

Estudo longitudinal prospectivo de base comunitária conduzido em hospital universitário.

**MÉTODOS:**

Dados sobre AVC baseados em eventos fatais e não fatais foram avaliados, incluindo dados sociodemográficos, mortalidade e preditores, por meio de regressão logística e análises de sobrevida.

**RESULTADOS:**

O subtipo de AVC foi melhor definido no ambiente hospitalar do que na comunidade local. Na fase hospitalar, cerca de 70% eram eventos primários e do subtipo isquêmico. Entre os fatores de risco cerebrovascular, a frequência de ingestão de álcool foi mais alta no AVC hemorrágico comparado com o isquêmico (35,4% *versus* 12,3%, P < 0,001). O risco de morte depois de AVC hemorrágico foi maior que o do AVC isquêmico e este gradiente foi máximo aos 10 dias após o evento (razão das chances, 3,31; intervalo de confiança (IC) de 95%: 1,55-7,05). Análise de sobrevivência em 4 anos com 665 casos de AVC primário (82,6% AVC isquêmico e 17,4% AVC hemorrágico) demonstrou taxa de sobrevida global de 48%. Aos 4 anos, maiores riscos de morte foram para casos de AVC isquêmico e pacientes analfabetos (*hazard ratio*, HR: 1,83; 95% IC: 1,26-2,68) ou com diabetes (HR:1,45; IC 95%: 1,07-1,97). ­Casos com depressão maior apresentaram pior sobrevida de 1 ano (HR: 4,60; IC 95%: 1,36-15,55).

**CONCLUSÃO:**

Em longo prazo, dados do EMMA fornecerão informações adicionais para planejamento de recursos destinados ao sistema de saúde público.

## INTRODUCTION

More than half of the global burden relating to cardiovascular disease (CVD) is concentrated in low and middle-income countries like Brazil.^1^ Although age-standardized rates of stroke mortality have declined over the last two decades, updated information from the Global Burden of Disease (GBD) study covering ­1990-2013 has shown that the absolute numbers of stroke cases have been increasing for both stroke subtypes, to reach around 10 million incident cases of stroke, 6.5 million deaths due to stroke and almost 26 million stroke survivors.^1^ Moreover, a rise in the absolute number of disability-adjusted life years (DALYs), mainly due to ischemic stroke (IS), which corresponded to 70% of all stroke cases, was observed over the same period. Lower incidences of IS for both sexes were observed in 2013, compared with 1990. However, higher incidence rates of IS among men than among women were still observed in 2013. Over the same period, no statistical differences in hemorrhagic stroke (HS) according to sex were noticed.^2^

In a global comparison, the stroke burden remains unequally distributed in developing countries.^1^ Particularly, stroke mortality rates in Brazil are the highest in Latin America.^3^^,^^4^^,^^5^^,^^6^ Brazilian data show that CVDs have ranked highest among mortality rates since the early 1960s and account for the highest proportion of hospitalizations. Although mortality rates should be interpreted with caution because of improvements in national statistics and the aging of the Brazilian population over recent decades, age-adjusted mortality rates relating to CVDs were seen to decline by around 20% from 2000 to 2011. Nonetheless, an increase in the overall number of CVD deaths was still reported (DATASUS, the data processing system of the Brazilian Ministry of Health). In 2011, CVDs were responsible for 31% of mortality and cerebrovascular diseases for 30% in this country. Similarly to global trends, CVD mortality rates in Brazil have been influenced by race, sex and other socioeconomic status (SES) characteristics. A greater decline in CVD mortality rates has also been observed among women than among men since 1996. Particularly, stroke mortality has declined by 3.6% and 3.3% per year among women and men, respectively.^7^ In addition, stroke mortality based on death certificate notifications is slightly higher among blacks than among mixed-race and white people.^7^ Data from the city of São Paulo (1996-2011) also followed the same trend in comparisons of both gender and family income. A greater decline in the stroke mortality rate was seen in relation to coronary heart disease (CHD) rates. The decrease in mortality was greater among women than among men and was inversely related to income, particularly for men.^8^

In addition to mortality data, the National Health Survey (PNS), which was a Brazilian community-based epidemiological survey with a nationally representative sample, provided estimates relating to around 2,231,000 stroke cases in 2013, of which 568,000 cases presented severe disabilities. The point prevalences were 1.6% and 1.4% for men and women, respectively. The prevalences of post-stroke disability were 29.5% for men and 21.5% for women. Although stroke prevalence rates were especially higher among older individuals without formal education who were urban dwellers than among individuals with high SES, the degree of stroke disability according to SES was not determined.^9^

Similarly to other data published worldwide, most research in developing countries, including in Brazil, has focused on the epidemiology of and therapeutic advances in CHDs rather than cerebrovascular diseases.^5^ Despite the undoubted importance of evaluating stroke epidemiology from a broad perspective, including prognostic factors and long-term mortality, there is still a lack of consistent data from developing countries.^10^ and the majority are from developed countries.^10^^,^^11^^,^^12^^,^^13^ Most previous stroke surveillance surveys in Brazil have been based on community and population-based studies restricted to one year at most.^14^^,^^15^ In fact, few authors have assessed long-term stroke survival or post-stroke disability, or even prognostic risk factors among stroke survivors in Brazil.^16^^,^^17^^,^^18^ One of these long-term stroke cohorts is the Study of Stroke Mortality and Morbidity in Adults (EMMA Study), which is an ongoing stroke surveillance survey in which the main objectives are to report on headline mortality rates and to monitor disability and prognostic risk factors among survivors living in a low-income area of the city of Sao Paulo, Brazil.^17^^,^^19^^,^^20^^,^^21^

## OBJECTIVE

Here, we describe this Brazilian initiative, focusing on concepts and the main findings regarding stroke burden, including mortality and prognostic risk factors among stroke survivors living in a low-income area who were enrolled in the EMMA cohort between 2006 and 2014.

## METHODS

### Design, ethics and setting

This is an ongoing prospective community-based stroke cohort study conducted at the Hospital Universitário, University of São Paulo (Universidade de São Paulo, USP).

The institutional review board of USP's university hospital approved the main study, and also ancillary studies linked to the EMMA cohort.

### Population

We evaluated stroke distribution and mortality within all three settings of the WHO STEPS stroke surveillance approach, in a low-income population living in the Butantan area. This area comprises six districts on the western side of São Paulo, with an estimated population of 424,377 (2009), of whom only 12% are over 60 years of age. Among these six districts, the proportion of households with a family income less than or equal to five monthly minimum wages (2000, National Census data) ranges from 13.1% to 40.8%. This range is narrower than in other districts of this city (6.4-60.3%). Cardiovascular diseases account for 40% of all deaths in Butantan and São Paulo, and stroke mortality represents one quarter of all vascular deaths. The proportion of violent deaths during the last 10 years was slightly lower in the Butantan area (4.8%) than in the city overall (5.9%).

In Butantan, there are 16 primary care facilities, seven with an emergency room. The only hospital in the area is the university hospital (Hospital Universitário) of the University of Sao Paulo (USP), which is a community hospital with 260 beds in which STEP 1 was implemented.^19^ This hospital supports emergencies from primary care units and paramedic ambulances and it is responsible for 80% of the hospitalizations of people living in this location. The center for neurological referral from this community facility is Hospital das Clinicas, which is a tertiary-care hospital located 8 km away. The primary care units are affiliated to the University, which also manages both hospitals.

### EMMA registry in the different settings

#### WHO STEPS stroke approach within the EMMA cohort

The data collection for the EMMA study was initially based on the WHO manual Stepwise Approach to Surveillance.^22^ The methodology for case ascertainment data and management of the entire STEPwise method is described elsewhere.^19^

In brief, STEP 1 (hospital phase), which is still ongoing, was started at USP's university hospital among patients who had neurological symptoms, fulfilled the initial criteria and agreed to participate in EMMA. The main study enrolled its first participant in April 2006 and its last one in September 2014. In this phase, we described the main objectives of the WHO project and some preliminary data, comprising evaluation of hospitalized events and including sociodemographic data (i.e. name, gender, age, race, income, educational level and occupation), acute stroke information regarding stroke recurrence, date and time of onset of stroke symptoms, hospitalization, history of traditional risk factors associated, medical treatment, neurological functionality (modified Rankin scale) and discharge status.^19^

For STEP 2 (fatal events in the community), which began in November 2006 and ended in 2007, the WHO methodology for cerebrovascular disease was applied in order to investigate cases that evolved to death through application of questionnaires that had previously been set up by WHO plus additional information relating to local realities.^19^ Mortality data was obtained from the city of São Paulo's health statistics system (PRO-AIM, "Programa de Aprimoramento das Informações de Mortalidade", i.e. the program for improving mortality information). The objective was to identify the set of characteristics that might make it possible to quantify and qualify deaths that occurred within the university hospital's catchment area. In this approach, information on reported deaths was collected according to health areas determined by the Municipal Health Department of the city of São Paulo, taking into account the respective area covered by each primary care unit. From this information, a protocol for action towards this disease was recommended.

STEP 3 (non-fatal events in the community) started in February 2008 and finished in May 2008. The community area was previously delimited through a public family healthcare program at one primary care unit within the university hospital's catchment area (at Jardim São Jorge). A potential total number of 4,725 subjects older than 35 years of age was estimated for this first part. Trained interviewers administered the screening instrument, asking each family member to answer symptom questions and to perform simple physical tasks.^23^^,^^24^ All participants who had been screened positive for events suggestive of stroke in the past were invited to answer an individual questionnaire that asked for information similar to that of the STEP 1 questionnaire. All of these individuals were classified according to their clinical diagnosis and their clinical and tomographic diagnoses of stroke. In relation to this last item, the stroke diagnoses of a subset of the patients were validated by a neurologist.^23^^,^^24^

#### Additional actions within the EMMA cohort

In addition to the STEPS stroke approach, we implemented other tools to investigate short and long-term mortality and the prognostic risk factors associated with survival, over the course of the follow-up on the EMMA cohort.^17^^,^^20^^,^^21^^,^^25^^,^^26^^,^^27^^,^^28^^,^^29^^,^^30^ An extension of the EMMA protocol, which was developed in collaboration with this researcher's time spent within the main study, was also implemented in a municipality of the Amazon region, in northern Brazil.^31^

#### Mortality and prognosis 

Vital status was investigated periodically by means of a hot-pursuit strategy using telephone contacts and medical registries during the follow-up. Particularly with regard to the main cohort at USP's university hospital in São Paulo, we doubled-checked all the mortality data through collaboration with the municipal statistics system (PRO-AIM), the data analysis system of the state of São Paulo (Fundação Sistema Estadual de Análise de Dados, SEADE) and the Brazilian Ministry of Health offices, every year. The reported causes of death on death certificates were transformed into medical codes in accordance with the Tenth Revision of the International Classification of Diseases (ICD-10).^31^ Ultimately, mortality was categorized as all-cause, cerebrovascular or cardiovascular. Here, we report data from previous EMMA publications based on all-cause mortality.

In the EMMA study, the mortality analyses included evaluation of case-fatality rates at 10, 28 and 180 days and survival analyses one year after the acute event, with exploration of some prognostic risk factors.^17^^,^^20^^,^^21^ Short-term mortality (10 and 28-day case-fatality rates) was compared with other stroke registries in other Brazilian cities located in the northeastern region (João Pessoa and Natal).^26^

Regarding prognosis over the course of the follow-up, we implemented an extended evaluation of cognitive impairment, using a specific validated questionnaire (Modified Telephone Interview for Cognitive Status, TICS-M) on a subsample of EMMA survivors, three months after the index event.^27^ In addition, post-stroke depression (PSD) after the acute phase and its influence on one-year survival was evaluated among stroke survivors, who answered a questionnaire on depression, the Patient Health Questionnaire (PHQ-9), by means of telephone interviews conducted one to three months after the acute event.^28^

Furthermore, we assessed an experimental open-case series to ascertain the effect of transcranial direct current stimulation (tDCS). This is a novel treatment that may improve clinical outcomes from PSD, which is traditionally refractory to pharmacotherapy, among stroke patients with aphasia during the first year after their stroke.^29^

#### Multicenter evaluation

An extended evaluation based on the original EMMA protocol (19) was also developed in the city of Coari, located in the Brazilian Amazon region. In this municipality, cerebrovascular prevalences were compared between the urban zone and rural riverbank areas of the municipality, between May and October 2011.^30^

#### Stroke ascertainment

We ascertained all consecutive cases of potential acute stroke events in the hospital, including first-ever and recurrent events. All patients older than 18 years of age were eligible for STEP 1. The WHO definition of stroke was used, i.e. "a focal (or at times global) neurological impairment of sudden onset, and lasting more than 24 hours (or leading to death), of presumed vascular origin."^22^ Histories of stroke were based on information from patients, caregivers or hospital records. When it was not possible to obtain information, the item was coded as "incomplete data". Stroke diagnoses were validated by a medical practitioner and supported by non-contrasted computed tomography (CT) scans. We used the codes of chapter I of ICD-10 to categorize stroke according to the following subtypes: ill-defined or unspecified stroke (ICD-10:I64), intracerebral hemorrhage (ICD-10:I61), cerebral infarction (ICD-10:I63), late effects of cerebrovascular diseases (ICD-10:I69) and subarachnoid hemorrhage (ICD-10:I60). All suspected stroke cases were also categorized as previous stroke (recurrent incidence of stroke) or no previous stroke (first-ever incidence of stroke), through access to medical records. 

All data collection was performed by trained interviewers and medical researchers in accordance with the instructions in the STEPS stroke manual. Quality control was assured through cross-checking the information, which was done by three medical coordinators of the EMMA study.

#### Statistics

The main baseline findings were reported as absolute and relative frequencies^19^^,^^20^^,^^21^^,^^25^^,^^26^ or as prevalence rates^23^ for categorical variables; and as parametric or nonparametric test results, in accordance with the distribution of continuous variables in each subsample that had been evaluated in previous publications.^19^^,^^20^^,^^21^^,^^23^^,^^24^^,^^25^^,^^26^^,^^27^^,^^28^^,^^29^^,^^30^

In the EMMA study, the mortality analyses included evaluation of case-fatality rates at 10, 28 and 180 days, and at one year, calculated by means of the chi-square test and logistic regression, using the odds ratio (OR) and 95% confidence interval (95% CI).^25^^,^^26^ One year after the index event, survival analyses were performed by means of Kaplan-Meier survival curves and Cox regression models, using the hazard ratio (HR) and 95% CI, to investigate predictors for long-term mortality, such as educational level, gender and depression.^17^^,^^20^^,^^21^ Further details on the statistical analyses in each previous publication are summarized in Table 1.

**Table 1:** Executive summary of previous publications from the Study of Stroke Mortality and Morbidity in Adults (EMMA Study), 2006-2014

Study	Objectives	Methodology	Main findings and conclusions
Goulart et al., 2010^19^	To evaluate stroke epidemiology based on WHO STEPS stroke surveillance in São Paulo, Brazil: STEP 1: hospitalized fatal and non-fatal events STEP 2: fatal events in the community STEP 3: stroke survivors in the community.	Design/population: cross-sectional evaluation based on the WHO Stepwise Approach to Stroke Surveillance: STEP 1: hospital-based data comprising fatal and nonfatal stroke cases. STEP 2: stroke-related mortality data in the community using WHO questionnaires. STEP 3: questionnaire determining stroke prevalence, applied door-to-door within a family-health program in the neighborhood. Exclusion criteria: people who did not belong to the reference area. Statistics: frequencies, prevalence (95% CI), case-fatality rates according to CBV.	STEP 1: 682 CBV cases ≥ 18 years: 472 incident cases, presented with CBV (84.3% with IS and 85.2% with first-ever stroke) from April 2006 to May 2009. STEP 2: 256 deaths from stroke were identified during 2006-2007. 44% of the deaths were unspecified stroke, 1/3 were IS, and 1/4 were HS. STEP 3: 577 subjects ≥ 35 years were screened at home, and 243 cases of stroke survival were diagnosed via a questionnaire, validated by a board-certified neurologist.
Abe et al., 2010^24^	To validate a questionnaire for evaluating individuals with stroke symptoms in the EMMA study, São Paulo, Brazil.	Design/population: cross-sectional evaluation in a sample from all households in the coverage area of a primary healthcare unit in Butantan. Household members ≥ 35 years answered a stroke symptom questionnaire addressing limb weakness, facial weakness, speech problems, sensory disorders and impaired vision and 36 participants were randomly selected for a complete neurological examination (gold standard). Exclusion criteria: people who did not belong to the reference area and were not able to give responses to survey. Statistics: frequencies, sensitivity, specificity, PPV, NPV, positive and negative LH.	Questionnaire properties: sensitivity 72.2%, specificity 94.4%, PPV 92.9% and NPV 77.3%. LR+ was 12.9, LR- was 0.29. Limb weakness was the most sensitive symptom, and speech problems were the most specific. The stroke symptom questionnaire is a useful tool and can be applied by trained interviewers with the aim of identifying community-dwelling stroke patients, through the structure of the Family Health Program.
Abe et al., 2011^23^	To verify the prevalence of stroke in a deprived neighborhood in São Paulo, Brazil and compared it with other surveys worldwide.	Design/population: cross-sectional evaluation using questionnaire with six questions concerning limb and facial weakness, articulation, sensory disturbances, impaired vision and past diagnosis of stroke was completed door-to-door in a well-defined area of 15,000 people. Questionnaires were considered positive when a participant answered two or more questions about stroke symptoms or when the presence of stroke was confirmed by a physician, or when at least three questions had positive findings, even if not confirmed by a doctor. Exclusion criteria: people who did not belong to the reference area and were not able to give responses to survey. Statistics: prevalence rates (95% CI).	A total of 243 people initially screened positive for stroke. Age-adjusted prevalence rate for men was 4.6% (95% CI: 3.5-5.7). For women, the prevalence rate was 6.5% (95% CI: 5.5-7.5); when considering only one question, the rate was 4.8% (95% CI: 3.9-5.7). Most commonly reported symptoms were limb weakness and sensory disorders.
Goulart et al., 2012^25^	To identify case-fatality rates up to one year in a community hospital in São Paulo, Brazil.	Design/population: cross-sectional evaluation of all patients with first-ever stroke seeking acute care at the hospital's emergency ward between April 2006 and December 2008, to verify early and late case fatality according to stroke subtype. We used years of formal education as a surrogate for socioeconomic status. Exclusion criteria: people who did not belong to the reference area; those who did not receive first acute treatment at the community hospital; and cases of recurrent stroke. Statistics: frequencies, case-fatality rates and OR from 10 days to 1 year.	Out of 430 first-ever stroke events, 365 (84.9%) were IS. After 1 year, we reported 108 deaths (86 of IS; 22 of HS). Age-adjusted case-fatality rates for IS and HS were: 6.0% versus 19.8% at 10 days, 10.6% versus 22.1% at 28 days, 17.6% versus 29.1% at 6 months and 21.0% versus 31.5% at 1 years. Low education was a predictor of death at 6 months (OR: 4.31; 95% CI: 1.34-13.91) and 1 years (OR: 4.21; 95% CI: 1.45-12.28) particularly in IS cases.
Fernandes et al., 2012^21^	To evaluate the functional dependence of stroke survivors in the EMMA study.	Design/population: cross-sectional evaluation of ischemic stroke individuals who survived after acute phase (after 28 days) in subsample from EMMA using the modified Rankin scale at 28 days and 6 months. Exclusion criteria: people who did not belong to the reference area; those who did not receive first acute treatment at the community hospital; HS; and recurrent stroke. Statistics: frequencies, case-fatality rates and OR from 10 days to 1 year.	Among 355 survivors from first-ever IS (mean age: 67.9 years), 40% had some functional dependence at 28 days and 34.4% had some functional dependence at 6 months. Most predictors of physical dependence were identified at 28 days, and these comprised low education (OR: 3.7; 95% CI: 1.60-8.54) and anatomical stroke location (total anterior circulation infarct; OR: 16.9; 95% CI: 2.93-97.49).
Fernandes et al., 2012^26^	To evaluate early stroke case-fatality rates in three hospitals in three distinct cities located in two macroregions of Brazil.	Design/population: cross-sectional evaluation of 10 and 28-day case-fatalities in stroke registries in São Paulo, João Pessoa and Natal. Exclusion criteria: people who did not belong to the reference area; those who did not receive first acute treatment at the hospitals; and cases of recurrent stroke. Statistics: frequencies and case-fatality rates at 10 days, 28 days and 6 months after acute event.	Out of 962 first-ever events (mean age: 68.1 years; 53% men), 83.6% were classified as IS and 16.4% as HS. Overall, the case-fatality rates and 95% CI for HS were higher than for IS, both at 10 days [12.3%; (95% CI: 7.2-17.4) versus 7.0% (95% CI: 5.3-8.8) and at 28 days (19.8%; 95% CI: 13.6-26.0 versus 11.1%; (95% CI: 8.9-13.3)].
Barros et al., 2013^27^	To evaluate the distribution of stroke hospital admissions (weekdays or weekend) and their respective prognosis based on a sample from the EMMA study.	Design/population: prospective evaluation of all consecutive stroke cases between April 2006 and December 2008, with subsequent 1-year follow-up. Exclusion criteria: people who did not belong to the reference area; those who did not receive first acute treatment at the community hospital; and cases of recurrent stroke. Statistics: frequencies, case-fatality rates and OR from 10 and 28 days after acute event.	Out of 430 first-ever stroke cases in people ≥ 35 years old, no associations between frequencies of hospital admissions due to IS and HS and the specific day of the week on which the admission occurred were found. However, 10 and 28-day case-fatality rates were higher in HS cases admitted at the weekend. It was found that HS admitted on weekends had a worse survival rate (50%) than those admitted during weekdays (25.6%; P log-rank = 0.03). Multivariate HR was 2.49 [95% CI: 1.10-5.81; P trend = 0.03] for risk of death at the weekend compared with weekdays for HS cases, but no difference in survival was observed for IS cases.
Goulart et al., 2013^17^	To identify prognostic factors associated with long-term stroke survival among IS and HS first-ever stroke cases in the EMMA study.	Design/population: prospective evaluation on consecutive IS and HS stroke cases in a community hospital in São Paulo, Brazil. Cardiovascular risk factors and sociodemographic characteristics (age, gender, race and educational level) were evaluated as prognostic factors. Exclusion criteria: people who did not belong to the reference area; those who did not receive first acute treatment at the community hospital; and cases of recurrent stroke. Statistics: Kaplan-Meier survival and crude and multiple Cox proportional hazard models (with HR) were performed.	Among 665 first-ever stroke cases, we found a lower survival rate among HS cases than among IS cases at the end of 4 years of follow-up (52% versus 44%; P = 0.04). The risk of death was highest among people with IS and without formal education (HR: 1.83; 95% CI: 1.26-2.68) and with diabetes (HR: 1.45; 95% CI: 1.07-1.97). Age had equal influence on the high risk of poor survival, regardless of stroke subtype. In addition, HS, low education and diabetes were significant independent predictors of poor long-term survival.
Fernandes et al., 2014^31^	To determine the CBV prevalence in a town in the Brazilian Amazon region, comparing urban versus rural population in the same municipality.	Design/population: cross-sectional evaluation of CBV to calculate prevalence rates among 6,216 residents ≥ 35 y old in the town of Coari, Amazonas, using a screening questionnaire, the Stroke Symptom Questionnaire. CBV prevalence rates (PRs) from the door-to-door survey were calculated according to the location of the home. Exclusion criteria: people who did not belong to the reference area and were not able to give responses to survey. Statistics: prevalence rates (95% CI).	There were 4,897 respondents in the urban area and 1,028 in the rural area. The crude prevalence of stroke was 6.3% in the rural area and 3.7% in the urban area, regardless of age and sex. Among stroke cases, people in the rural area were those with less access to medical care in comparison with the urban area (32.1% versus 52.5%; P = 0.01), and there was a positive association between the rural area and no medical care (PR: 1.33; 95% CI: 1.03-1.71), independently of age, sex, education and functional impairment.
Baccaro et al., 2015^28^	To examine the psychometric properties of the Brazilian version of the Modified Telephone Interview for Cognitive Status (TICS-M) for cognitive impairment in a post-stroke subsample from the EMMA study.	Design/population: validation study. Original version of the TICS-M was translated and adapted into Brazilian-Portuguese language, and applied to 30 non-clinical subjects. After that, two trained researchers applied TICS-M to 61/73 EMMA participants who completed the follow-up: (i) personal interview 6 months after stroke; (ii) telephone interview 1 week after the first evaluation; and (iii) telephone interview 2 weeks after the first evaluation. Exclusion criteria: aphasia or other clinical conditions that made the interview impossible. Statistics: Reliability (test-retest) using Pearson's correlation, ICC and Cronbach's alpha coefficient. ROC analysis using the MMSE was used as a comparison. Structural validity of TICS-M was assessed through PCA in relation to 103 individuals (30 non-clinical and 73 stroke patients).	Reliability and ICC ranged from 0.87 to 0.97 across the evaluations. Cronbach's alpha was 0.96. PCA analysis extracted three meaningful domains: working memory, recall memory and orientation. Best cutoff point for screen cognitive impairment was 14 out of 15 (91.5% sensitivity; 71.4% specificity). The area under the curve was 0.89.
de Mello et al., 2016^29^	To evaluate the influence of major depression disorder (MDD) on long-term survival in a subsample of EMMA participants.	Design/population: prospective evaluation of IS and HS cases. The Patient Health Questionnaire (PHQ-9) for MDD was applied 30 days after index event and periodically during 1-year follow-up. All participants were able to answer telephone interview. Exclusion criteria: aphasia or other clinical conditions that made the interview impossible. Statistics: Kaplan-Meier survival and crude and multiple Cox proportional hazard models.	Among 164 (85.9%) subjects with IS and 27 (14.1%) with HS, overall incidence of MDD was 25.1% during 1 year of follow-up, regardless of stroke subtype or recurrence. The peak rate of MDD was more than 1 month post-acute event. Post-stroke MDD was associated with lower survival after 1 year of follow-up (85.4% with MDD versus 96.5% without MDD; log-rank P = 0 .006). A higher and independent risk of all-cause mortality among those who developed MDD compared with participants without MDD was also detected (HR: 4.60; 95% CI: 1.36-15.55; P = 0 .01).
Valiengo et al., 2016^30^	To evaluate the safety and efficacy of tDCS for patients with post-stroke depression (PSD) and with aphasia post-stroke.	Design/population: open-label study on PSD depression diagnosed by means of Aphasic Depression Questionnaire (SADQ) and the Aphasic Depression Rating Scale (ADRS), to evaluate the severity of PSD in four first-ever stroke cases from October 2012 to August 2014. Diagnoses of PSD and aphasia were confirmed by a psychiatrist and a speech-language pathologist, respectively. All eligible individuals (subsample from EMMA) received 10 sessions (once a day) of bilateral tDCS to the dorsolateral prefrontal cortex (DLPFC) and two additional sessions after two and four weeks (total of 12 sessions). Exclusion criteria: non-confirmed PSD and aphasia. Statistics: patients' scores from the ADRS and SADQ were subjected to one-way analysis of variance (ANOVA) with time (baseline, week 2, week 4, week 6) as the within-subject factors. Post-hoc comparisons were carried out using Bonferroni test. Additionally, the partial Eta squared (pη2) was calculated for each ANOVA.	Among the four females evaluated, all exhibited improvement in depression after tDCS [decreases in SADQ (47.5%) and in ADRS (65.7%)]. This improvement was maintained four weeks after the treatment. In this preliminary open-label study conducted on four PSD patients with aphasia, bilateral tDCS over the DLPFC was shown to induce a substantial mood improvement; tDCS was safe and well tolerated by every patient.

CBV = cerebrovascular disease; WHO = World Health Organization; IS = ischemic stroke; HS = hemorrhagic stroke; 95% CI = 95% confidence interval; PPV = positive predictive value; NPV = negative predictive value; OR = odds ratio; HR = hazard ratio; ICC = Cronbach's intraclass correlation coefficient; ROC = receiver operating characteristic curve; PCA = principal-component analysis; tDCS = transcranial direct current stimulation.

## RESULTS

Previous publications from the EMMA study are summarized in Table 1.

### Main findings from STEPS within the EMMA study

The first published data from EMMA study reported on 682 stroke cases out of 1,023 cases of cerebrovascular disease (66.6%). The participants were over 18 years of age and their acute stroke event was confirmed through medical diagnosis and CT within the first 24-48 hours, upon hospital admission. All of them were attended at the university hospital's emergency care sector and were enrolled in EMMA (STEP 1) between April 2006 and May 2009.^19^

During the surveillance of fatal events within the community (STEP 2), 256 deaths due to stroke were identified over a 12-month follow-up period. The primary cause of death (causa mortis) according to stroke subtype showed that 30.5% of the deaths were due to IS, 26.6% were due to HS and 43% had an unspecified form of stroke as the primary cause.^19^

The initial screening of non-fatal stroke cases in the community (STEP 3) included 4,446 individuals living in the reference area of USP's university hospital, near to the Jardim São Jorge primary care unit, which is located in the western area of the city of São Paulo. Among these individuals, 618 (13.7%) were not found, 204 (4.5%) refused to participate in the study and 13 (0.29%) were incapable of answering the questions. In total, 3,661 individuals (81.4%) answered a familial screening questionnaire, and 582 of them were identified as having screened neurologically positive, based on additional information relating to their treatment, disability and neurological recovery after stroke. A total of 577 subjects answered the final questionnaire, of whom 243 were screened positive for stroke, based on a questionnaire, and were validated by a board-certified neurologist.^23^^,^^24^ The age-adjusted prevalence rate for men was 4.6% (95% CI: 3.5-5.7). For women, the prevalence rate was 6.5% (95% CI: 5.5-7.5) and, when considering only one question, the rate was 4.8% (95% CI: 3.9-5.7). The most commonly reported symptoms were limb weakness and sensory disturbances. Hypertension and heart disease were very frequent conditions associated with previous stroke.^23^

In all settings, most of the participants were white and married, and had low education (1-7 years). We observed that most of the subjects in STEP 1 and 2 were older (mean ages: 66 and 74 years, respectively) than those who participated in STEP 3 (50.7% of subjects were of ages ranging from 45 to 64 years). Regarding the respondents' gender, we observed that in STEP 3, more females participated in the study (59.4% in STEP 3, 49.2% in STEP 2 and 45.3% in STEP 1).

### Short and long-term mortality during extended follow-up

Regarding the mortality rates from the hospital registry, we evaluated case-fatality rates from ten days to one year among all consecutive patients with first-ever stroke who sought acute care at the USP university hospital's emergency service and were enrolled in the EMMA study between April 2006 and December 2008. Among 430 first-ever stroke events, 365 (84.9%) were IS and 65 (15.1%) were HS.^25^ Among cerebrovascular risk factors, the frequency of alcohol intake was higher in hemorrhagic stroke (HS) than in ischemic stroke (IS) cases (35.4% versus 12.3%, P < 0.001). After one year, we found that 108 deaths had occurred (86 cases of IS and 22 of HS). The age-adjusted case fatality rates for IS and HS were 6.0% versus 19.8% at 10 days, 10.6% versus 22.1% at 28 days, 17.6% versus 29.1% at six months, and 21.0% versus 31.5% at one year. Illiteracy or no formal education was a predictor for death at six months (OR: 4.31; 95% CI: 1.34-13.91) and one year (OR: 4.21; 95% CI: 1.45-12.28) among patients with ischemic stroke. It was also a predictor at six months (OR: 3.19; 95% CI: 1.17-8.70) and one year (OR: 3.30; 95% CI: 1.30-8.45) for all stroke patients. Other variables, including previous cardiovascular risk factors and acute medical care, did not change this association to a statistically significant degree. In conclusion, case fatality, particularly up to six months, was higher in cases of hemorrhagic stroke, and lack of formal education, particularly among IS cases, was associated with increased stroke mortality.^25^

Our early case-fatality rates were also compared with other stroke registries located in general hospitals in the northeastern region of Brazil (cities of João Pessoa and Natal).^26^ Out of 962 first-ever events recorded in three centers, the proportions of ischemic-to-hemorrhagic cases were maintained at 5:1, as we previously observed in our single-center analysis at USP's university hospital, where the EMMA study was set up.^25^

Additional long-term mortality data, including survival analyses from April 2006 to December 2010, were used to evaluate 665 first-ever stroke cases, of which 545 (82.6%) were IS and 116 (17.4%) were HS during the four-year follow-up. We found an overall survival rate of 48% (mean survival of 40 months). Again, we confirmed that lack of formal education and diabetes were independent predictors of poor survival, particularly among IS subjects during long term-follow-up^15^ (Table 1).

### Cognitive impairment

Cognitive status three months after acute stroke was evaluated during the follow-up as an additional action relating to post-stroke disabilities. In this context, we previously adapted and validated the Brazilian version of the TICS-M for cognitive impairment among post-stroke patients, in a subset of EMMA participants, using the Mini-Mental State Examination (MMSE) as the comparison.^28^ We found that cognitive impairment was present in 22.9% of the individuals, post-stroke. The test-retest reliability and intraclass correlation from TICS-M were found to be good, with coefficients ranging from 0.87 to 0.97 across the evaluations. Principal-component analysis extracted three meaningful domains: working memory, recall memory and orientation. The best cutoff point for screening for cognitive impairment was 14 out of 15 (91.5% sensitivity and 71.4% specificity), based on MMSE as the comparison. The area under the curve was 0.89 and, in the end, we concluded that the Brazilian version of the TICS-M was a reliable, stable and homogeneous instrument for screening for cognitive impairment among stroke patients.^28^

### Post-stroke depression

In a subsample of 191 EMMA participants who reported their depressive status using PHQ-9, one to three months after the acute event, we found that 164 (85.9%) had suffered IS and 27 (14.1%), HS. Among these, the overall incidence of major depression disorder was 25.1% during the one-year follow-up, regardless of stroke subtype. The peak rate of major depression subsequent to the acute event was more than one month afterwards. We observed that there was a lower survival rate among individuals who developed post-stroke major depression disorder than among those who did not develop this condition, after one year of follow-up (85.4% versus 96.5%; log rank P = 0.006). After multiple analysis, we found that there continued to be higher risk of all-cause mortality among those who developed major depression disorder than among participants without major depression disorder (HR: 4.60; 95% CI: 1.36-15.55; P = 0.01), thus suggesting that incident major depression disorder is a potential marker for poor prognosis, one year after stroke.^29^

### tDCS in post-stroke depression (PSD) and aphasia

The sample comprised four females (mean age: 48 years) with aphasia after stroke who developed the onset of post-stroke depression after the index event (mean time elapsed: six months). The treatment was well tolerated by all these patients and no adverse effects were observed. For the Aphasic Depression Rating Scale (ADRS), the main effects of time reached significance: post-comparisons showed significant reductions in the patients' scores only at week 6 (5.5), in comparison with baseline (16; P < 0.001), week 2 (12.75; P < 0.01) and week 4 (11.75; P < 0.02). For the Aphasic Depression Questionnaire (SADQ), the significant main effects of time were reductions of patients' scores from baseline (56.25) to week 2 (38.5; P < 0.001), week 4 (35.5; P < 0.001) and week 6 (29.75; P < 0.0001).^30^

### Extended EMMA initiative

In the area studied in Coari, 6,216 residents over 35 years of age were interviewed using a screening questionnaire, the Stroke Symptom Questionnaire. From this door-to-door surveillance, cerebrovascular prevalence rates (PR) were calculated according to the location of the home.^31^ The total numbers of respondents were 4,897 in the urban area and 1,028 in the rural area. The crude prevalence rate (PR) of stroke was 6.3% in the rural area and 3.7% in the urban area, regardless of age and sex. As expected, lower levels of medical care were observed in the rural area than in the urban area (32.1 versus 52.5%, P = 0.01). There was a positive association between living in the rural area and no medical care for stroke (PR: 1.33; 95% CI: 1.03-1.71), regardless of SES.^31^

## DISCUSSION

Since 2006, unified data provided through the EMMA study have demonstrated trends regarding stroke surveillance in three spheres of investigation (hospital, official mortality data and community sources).^19^^,^^23^ The data have also shown potential risk factors and disability and mortality statistics based on case-fatality and survival rates in this low-income population over the course of four-year follow-up.^17^^,^^20^^,^^21^^,^^25^^,^^26^

At first view, the demographic characteristics among the EMMA participants were similar in the three STEPS, except for age and sex. In the community (STEP 3), we found younger survivors and more females than in other settings.^19^ As expected, information on stroke subtype was better defined in the hospital setting (STEP 1) than in the community (STEP 3).^19^ Among the cases included in the hospital phase, about 70% were confirmed as first-ever stroke and ischemic subtype (ratio of hemorrhagic-to-ischemic cases of 1:6) during the period 2006-2009.^25^ We noticed that there was a slight increase in the proportions of hemorrhagic-to-ischemic cases. to 1:4, by adding one year of follow-up (2006-2010).^17^ In addition to aging, regular alcohol consumption was closely associated with intracerebral hemorrhage.

Comparisons across epidemiological studies on stroke worldwide are difficult because of divergences of methodology, especially regarding the study sample (hospital or community or population-based data), the criteria used for ascertaining cases and the stroke subtype enrolled. Nevertheless, the EMMA study, which used a stroke cohort based on a low-income community on the western side of the city of São Paulo, had results that were in accordance with those from most population-based studies.^32^ In addition, the extension of the EMMA study to a community in the Brazilian Amazon region confirmed that the prevalence of stroke in rural areas is higher than in urban areas.^31^

A systematic review conducted on 56 population-based studies, including Brazilian data,^14^ which reported stroke incidence and case-fatality from 1970 to 2008 in low to middle-income countries, found that the proportion of ischemic stroke ranged from 54% to 85% and that the proportion of intracerebral hemorrhage ranged from 14% to 27% over the period from 2000 to 2008.^32^ As expected, hemorrhagic cases were more commonly detected in low-income countries.^32^ Although we found a slight increase in the proportion of incident cases of HS, compared with IS, over a four-year period, our rates were similar to those reported in developed countries.^32^ Regarding mortality, our case-fatality rate over a one-year period was 25% (ratio of ischemic to hemorrhagic stroke cases of 1:4). As expected, the risk of death due to HS was greater than the risk due to IS and this gradient reached its maximum at 10 days (OR: 3.31; 95% CI: 1.55-7.05). Low education was the main sociodemographic factor associated with higher risk of death, particularly among those with ischemic stroke. The influence of lack of education on mortality was markedly higher at 10 days.^25^

Our case rates for IS (85%) and HS (15%) were similar to those reported in other Brazilian studies.^14^^,^^33^ However, they differed from those reported in other countries in Latin America, such as Chile (72% for IS and 28% for HS)^33^ and were much more divergent from data from southern African (Mozambique), from where the highest rate of HS (46%) versus IS (56%) was reported.^34^

A comparison of our findings with those from two other Brazilian population-based studies showed that our study had a lower one-year overall case fatality rate than the rate reported in Matão (22.7% versus 30.9%)^14^ and a lower 180-day lethality rate than the rate in Joinville (overall stroke rate: 19.5% versus 25%; ischemic stroke rate: 17.6% versus 19%; and hemorrhagic stroke rate: 29.1% versus 49%).^33^

Overall, life expectancy within the first four years after stroke was about 50% in the EMMA study. Our cumulative survival rate for hemorrhagic stroke was 44%.^18^ The main determinants of poor survival up to four years were hemorrhagic stroke and lack of education for ischemic cases. Moreover, we found that diabetes was an independent predictor of all-cause mortality in a long-term follow-up on our study data.^18^

Regarding functionality, there was a slight decrease in the hospital phase of EMMA, from 40% at 28 days to 34% at six months after the acute event, particularly among individuals of low education level with IS.^21^ Reinforcing these findings, in EMMA STEP 3 (community level), the referral rate for rehabilitation services was roughly 25% for all participants within the community who were identified as presenting a previous history of stroke.^23^

Overall, our statistics revealed similar proportions for incident cases of ischemic and hemorrhagic stroke cases in comparison with developed countries. On the other hand, we continue to be behind developed countries in terms of decreasing the mortality rates. The case-fatality rates remain high and the survival rates remain poor in our setting. There is also a high degree of dysfunctionality among stroke survivors, particularly those with low SES, which is much more similar to the stroke pattern in developing countries.^1^ These findings reaffirm the trends recently reported by GBD in 2013, regarding the significant burden of stroke concentrated in developing countries. The impact on mortality rates, DALYs and years lived with disability (YLDs) that comes mainly from hemorrhagic stroke was shown to be significantly higher in developing than in developed countries. During the period from 1990 to 2013, the proportional contributions of deaths due to HS and IS increased by 1.8% and 2.2% in developing countries, respectively. Meanwhile, in developed countries, these rates decreased by 0.73% for HS and by 1.45% for IS.^1^

The EMMA study has some strengths. Although our cohort was not a population-based study, we based our data on a community area with low SES, located in a developing country. This may have contributed towards filling the gap in the information on stroke epidemiology. We developed an extended evaluation that included epidemiology, mortality and predictors associated with poor prognosis among individuals who survived the acute phase of the cerebrovascular event. We implemented a standard protocol in order to follow up our participants by means of telephone contacts and thus update the following data: vital status; functional disability; non-fatal outcomes such as hospitalization; recurrence of stroke or other CVD outcomes (heart failure and myocardial infarction); progression of depression; and cognition. All of these data will be available in the near future for prospective analysis.

The information acquired over the course of the follow-up was all double-checked by the medical researcher responsible, based on the patient's medical records and additional examinations such as CT, echocardiography and electrocardiogram for the main study. The mortality information was all confirmed through official death certificates provided by the local health statistics departments at all centers involved in the stroke surveillance. Thus, mortality specified as due to cerebrovascular or cardiovascular causes will be available for survival analyses.

Other than the EMMA cohort, only a few Brazilian studies have reported on the big picture of stroke epidemiology, including long-term follow-up with its admixture of outcomes.^16^^,^^18^^,^^35^

Finally, the contribution of the EMMA cohort related to knowledge of stroke epidemiology in hospital and community settings, thereby enabling comparisons across developing countries that have applied the WHO methodology for stroke surveillance.^36^^,^^37^^,^^38^

Our main limitations related mainly to the initial data collection, which lacked acute neurological evaluation for quantifying stroke severity in most cases. We implemented the NIH Stroke Scale (NIHSS) from the outset of the study, but less than 10% of the cohort presented trustworthy data. In addition, we implemented some protocols to investigate post-stroke depression and cognitive impairment/dementia after 2010.

## CONCLUSIONS

Data provided by the EMMA cohort study have depicted stroke surveillance in three spheres of investigation (hospital, official mortality data and community sources). The foremost findings of high rates of post-stroke disability and mortality and poor long-term survival have mainly been influenced by low education levels so far, up to the four-year follow-up.

## References

[1] Feigin VL, Krishnamurthi RV, Parmar P (2015). Update on the Global Burden of Ischemic and Hemorrhagic Stroke in 1990-2013: The GBD 2013 Study. Neuroepidemiology.

[2] Barker-Collo S, Bennett DA, Krishnamurthi RV (2015). Sex Differences in Stroke Incidence, Prevalence, Mortality and Disability-Adjusted Life Years Results from the Global Burden of Disease Study 2013. Neuroepidemiology.

[3] Ribeiro AL, Duncan BB, Brant LC (2016). Cardiovascular Health in Brazil Trends and perspectives. Circulation.

[4] Lotufo PA (2015). Stroke is still a neglected disease in Brazil. Sao Paulo Med J.

[5] Lotufo PA, Goulart AC, Fernandes TG, Benseñor IM (2013). A reappraisal of stroke mortality trends in Brazil (1979-2009). Int J Stroke.

[6] Lotufo PA, Benseñor IM (2009). Stroke mortality in Brazil one example of delayed epidemiological cardiovascular transition. Int J Stroke.

[7] Lotufo PA, Benseñor IJ (2013). Raça e mortalidade cerebrovascular no Brasil [Race and stroke mortality in Brazil]. Rev Saúde Pública.

[8] Lotufo PA, Fernandes TG, Bando DH, Alencar AP, Benseñor IM (2013). Income and heart disease mortality trends in Sao Paulo, Brazil, 1996 to 2010. Int J Cardiol.

[9] Benseñor IM, Goulart AC, Szwarcwald CL (2015). Prevalência de acidente vascular cerebral e de incapacidade associada no Brasil Pesquisa Nacional de Saúde - 2013 [Prevalence of stroke and associated disability in Brazil: National Health Survey - 2013]. Arq Neuropsiquiatr.

[10] Yusuf S, Rangarajan S, Teo K (2014). Cardiovascular risk and events in 17 low-, middle-, and high-income countries. N Engl J Med.

[11] Andersen KK, Olsen TS, Dehlendorff C, Kammersgaard LP (2009). Hemorrhagic and ischemic strokes compared stroke severity, mortality, and risk factors. Stroke.

[12] Anderson CS, Jamrozik KD, Broadhurst RJ, Stewart-Wynne EG (1994). Predicting survival for 1 year among different subtypes of stroke Results from the Perth Community Stroke Study. Stroke.

[13] Pham TM, Fujino Y, Tokui N (2007). Mortality and risk factors for stroke and its subtypes in a cohort study in Japan. Prev Med.

[14] Minelli C, Fen LF, Minelli DP (2007). Stroke incidence, prognosis, 30-day, and 1-year case fatality rates in Matão, Brazil a population-based prospective study. Stroke.

[15] Lavados PM, Sacks C, Prina L (2007). Incidence, case-fatality rate, and prognosis of ischaemic stroke subtypes in a predominantly Hispanic-Mestizo population in Iquique, Chile (PISCIS project) a community-based incidence study. Lancet Neurol.

[16] Cabral NL, Longo A, Moro C (2011). Education level explains differences in stroke incidence among city districts in Joinville, Brazil a three-year population-based study. Neuroepidemiology.

[17] Goulart AC, Fernandes TG, Santos IS (2013). Predictors of long-term survival among first-ever ischemic and hemorrhagic stroke in a Brazilian stroke cohort. BMC Neurol.

[18] Cabral NL, Muller M, Franco SC (2015). Three-year survival and recurrence after first-ever stroke the Joinville stroke registry. BMC Neurol.

[19] Goulart AC, Bustos IR, Abe IM (2010). A stepwise approach to stroke surveillance in Brazil the EMMA (Estudo de Mortalidade e Morbidade do Acidente Vascular Cerebral) study. Int J Stroke.

[20] Goulart AC, Fernandes TG, Alencar AP (2012). Low education as a predictor of poor one-year stroke survival in the EMMA Study (Study of Stroke Mortality and Morbidity in Adults), Brazil. Int J Stroke.

[21] Fernandes TG, Goulart AC, Santos-Junior WR (2012). Nível de escolaridade e dependência funcional em sobreviventes de acidente vascular cerebral isquêmico [Educational levels and the functional dependence of ischemic stroke survivors]. Cad Saúde Pública.

[22] World Health Organization (2005). WHO STEPS Stroke Manual: the WHO STEPwise approach to stroke surveillance/Noncommunicable Diseases and Mental Health, World Health Organization.

[23] Abe IM, Lotufo PA, Goulart AC, Benseñor IM (2011). Stroke prevalence in a poor neighbourhood of São Paulo, Brazil applying a stroke symptom questionnaire. Int J Stroke.

[24] Abe IM, Goulart AC, Santos WR, Lotufo PA, Benseñor IM (2010). Validation of a stroke symptom questionnaire for epidemiological surveys. Sao Paulo Med J.

[25] Goulart AC, Bensenor IM, Fernandes TG (2012). Early and one-year stroke case fatality in Sao Paulo, Brazil applying the World Health Organization's stroke STEPS. J Stroke Cerebrovasc Dis.

[26] Fernandes TG, Goulart AC, Campos TF (2012). Taxas de letalidade precoce por acidente vascular cerebral em três registros hospitalares no nordeste e sudeste do Brasil [Early stroke case-fatality rates in three hospital registries in the Northeast and Southeast of Brazil]. Arq Neuropsiquiatr.

[27] Barros JB, Goulart AC, Alencar AP, Lotufo PA, Bensenor IM (2013). The influence of the day of the week of hospital admission on the prognosis of stroke patients. Cad Saude Publica.

[28] Baccaro A, Segre A, Wang YP (2015). Validation of the Brazilian-Portuguese version of the Modified Telephone Interview for cognitive status among stroke patients. Geriatr Gerontol Int.

[29] de Mello RF, Santos Ide S, Alencar AP (2016). Major Depression as a Predictor of Poor Long-Term Survival in a Brazilian Stroke Cohort (Study of Stroke Mortality and Morbidity in Adults) EMMA study. J Stroke Cerebrovasc Dis.

[30] Valiengo L, Casati R, Bolognini N (2016). Transcranial direct current stimulation for the treatment of post-stroke depression in aphasic patients a case series. Neurocase.

[31] Fernandes TG, Benseñor IM, Goulart AC (2014). Stroke in the rain forest prevalence in a ribeirinha community and an urban population in the Brazilian Amazon. Neuroepidemiology.

[32] Feigin VL, Lawes CM, Bennett DA, Barker-Collo SL, Parag V (2009). Worldwide stroke incidence and early case fatality reported in 56 population-based studies a systematic review. Lancet Neurol.

[33] Cabral NL, Goncalves AR, Longo AL (2009). Incidence of stroke subtypes, prognosis and prevalence of risk factors in Joinville, Brazil a 2 year community based study. J Neurol Neurosurg Psychiatry.

[34] Damasceno A, Gomes J, Azevedo A (2010). An epidemiological study of stroke hospitalizations in Maputo, Mozambique a high burden of disease in a resource-poor country. Stroke.

[35] Lavados PM, Sacks C, Prina L (2005). Incidence, 30-day case-fatality rate, and prognosis of stroke in Iquique, Chile a 2-year community-based prospective study (PISCIS Project). Lancet.

[36] Truelsen T, Heuschmann PU, Bonita R (2007). Standard method for developing stroke registers in low-income and middle-income countries experiences from a feasibility study of a stepwise approach to stroke surveillance (STEPS Stroke). Lancet Neurol.

[37] Dalal PM, Bhattacharjee M, Vairale J, Bhat P (2008). Mumbai stroke registry (2005-2006)--surveillance using WHO steps stroke instrument--challenges and opportunities. J Assoc Physicians India.

[38] Sridharan SE, Unnikrishnan JP, Sukumaran S (2009). Incidence, types, risk factors, and outcome of stroke in a developing country the Trivandrum Stroke Registry. Stroke.

